# Reply to a Comment Paper on the Published Paper by Canta, A. et al: “Calmangafodipir Reduces Sensory Alterations and Prevents Intraepidermal Nerve Fibers Loss in a Mouse Model of Oxaliplatin Induced Peripheral Neurotoxicity”—*Antioxidants* 2020, *9*, 594

**DOI:** 10.3390/antiox9090807

**Published:** 2020-09-01

**Authors:** Annalisa Canta, Alessia Chiorazzi, Eleonora Pozzi, Giulia Fumagalli, Laura Monza, Cristina Meregalli, Valentina A. Carozzi, Virginia Rodriguez-Menendez, Norberto Oggioni, Jacques Näsström, Paola Marmiroli, Guido Cavaletti

**Affiliations:** 1 Experimental Neurology Unit, School of Medicine and Surgery, University of Milano-Bicocca, Via Cadore 48, 20900 Monza, Italy; annalisa.canta@unimib.it (A.C.); alessia.chiorazzi@unimib.it (A.C.); e.pozzi18@campus.unimib.it (E.P.); g.fumagalli14@campus.unimib.it (G.F.); laura.monza@unimib.it (L.M.); cristina.meregalli@unimib.it (C.M.); valentina.carozzi1@unimib.it (V.A.C.); virginia.rodriguez1@unimib.it (V.R.-M.); norberto.oggioni@unimib.it (N.O.); guido.cavaletti@unimib.it (G.C.); 2 PledPharma AB, Grev Turegatan 11 C, 114 46 Stockholm, Sweden; jacques.nasstrom@pledpharma.se; 3 Department of Biotechnology and Biosciences, University of Milano-Bicocca, Piazza della Scienza 1, 20126 Milan, Italy

The comments sent by Stehr, Lundstom and Karlsson with reference to our article “Calmangafodipir reduces sensory alterations and prevents intraepidermal nerve fiber loss in a mouse model of oxaliplatin-induced peripheral neurotoxicity“ are very interesting, since they suggest possible mechanisms of action of the compound, which might contribute to its protective action.

However, we performed the study with a different aim, which was to search a thoroughly characterized mouse model for pathological evidence of calmangafodipir’s efficacy.

This approach was prompted by the clinical observation of its neuroprotective activity in oxaliplatin-treated patients, based on patient reported outcome (PRO) measures. The use of PROs is strongly supported by international regulatory agencies, but in clinical trials it is hardly possible to achieve pathological confirmation of the results. This might raise concerns over the objective extent of the perceived effect reported by patients and graded only using PRO measures.

As a consequence of our study design and its endpoint, we did not plan to collect biological samples that might be used for mechanistic studies on calmangafodipr activity, which might be the topic of specific new studies.

